# Adult child educational attainment and older parents’ psychosocial outcomes during the COVID-19 pandemic

**DOI:** 10.1186/s12889-024-19425-6

**Published:** 2024-07-31

**Authors:** Karla Renata Flores Romero, Yulin Yang, Sharon H. Green, Sirena Gutierrez, Erika Meza, Jacqueline M. Torres

**Affiliations:** 1 Department of Epidemiology & Biostatistics, UC San Francisco, 550 16th Street, San Francisco, CA 94158 USA; 2 Department of Demography, UC Berkeley, Berkeley, CA USA

**Keywords:** Socio-economic status, COVID-19 pandemic, Older adults, Mental health, Intergenerational influences

## Abstract

**Background:**

Older adults’ psychosocial outcomes during the COVID-19 pandemic have been inequitable by socio-economic status (SES). However, studies have focused solely on own SES, ignoring emerging evidence of the relationship between adult child SES and late-life health. We evaluated whether adult child educational attainment – a core marker of SES – is associated with older parents’ psychosocial outcomes during the pandemic.

**Methods:**

We used data from the Survey of Health, Aging, and Retirement in Europe (SHARE) 2004–2018 and the SHARE Corona Surveys (SCS) 2020 and 2021. We included 40,392 respondents ≥ 65 years who had pre-pandemic information on adult child educational attainment and self-reported psychosocial outcomes during the pandemic, including self-assessments of worsened psychosocial outcomes compared to the pre-pandemic period. We used generalized estimating equations with a Poisson distribution and a log link, adjusted for respondent and family-level characteristics, including respondents’ own educational attainment.

**Results:**

Older adults whose adult children averaged levels of educational attainment at or above (vs. below) their country-specific mean had a lower prevalence of feeling nervous (Prevalence Ratio [PR]: 0.94, 95% Confidence Interval [CI]: 0.90, 0.97), sad or depressed (PR: 0.94, 95% CI: 0.91, 0.98), and having sleep problems (PR: 0.94, 95% CI: 0.90, 0.97) during the pandemic. Additionally, higher adult child educational attainment was associated with a lower risk of perceiving worsened feelings of nervousness (PR: 0.95, 95% CI: 0.90, 1.01) and worsened sleep problems (PR: 0.91, 95% CI: 0.82, 1.01) as compared to the pre-pandemic. In stratified models, protective associations were observed only in countries experiencing “high” levels of COVID-19 intensity at the time of the survey. All of these results are derived from adjusted models.

**Conclusions:**

Adult child SES may have “upward” spillover effects on the psychosocial wellbeing of older parents during periods of societal duress like the pandemic.

**Supplementary Information:**

The online version contains supplementary material available at 10.1186/s12889-024-19425-6.

## Introduction

Older adults faced myriad adverse experiences during COVID-19 pandemic including due to the direct threats of infection and disease as well as disruption to social life. These and other challenges contributed to poorer mental wellbeing among older adults [1–5]. Nevertheless, studies have indicated that the psychosocial consequences of the COVID-19 pandemic have been inequitable across socio-economic strata, with the poorest outcomes concentrated among older adults with lower levels of education and other markers of socio-economic disadvantage [1, 3, 6–11]. However, this research has taken an individualistic approach by exclusively focusing on the impact of *own* socio-economic status, ignoring growing evidence that the socio-economic status (SES) of one’s family – and most notably, one’s adult children – can influence late-life psychosocial and related health outcomes [12–22]. To our knowledge, no study has specifically examined the impact of adult children’s SES on their parents’ health outcomes during the pandemic.

Research from the pre-pandemic period provides evidence of a plausible relationship between adult child SES and pandemic-era health, including mental health [12, 13, 16]. For example, numerous pre-pandemic studies have demonstrated a protective relationship between adult child educational attainment and psychosocial wellbeing among older adults, including fewer depressive symptoms and greater social engagement [23–25]. These relationships may be explained by the fact that adult children with higher SES may provide critical economic and non-economic resources to older parents, including direct financial transfers or resource sharing (e.g. sharing a household) [22]. During the pandemic, adult child economic resources may have been used to help older adults overcome pandemic-related challenges [26], like accessing technology needed to maintain social connections with non-resident family and friends. Higher SES among adult children has also been linked to lower levels of stress and worry for older parents (e.g. via reduced financial strain, improved relationship quality, or the exchange of social and instrumental support) as well as greater life satisfaction and quality of life [25, 27, 28]; these factors may underlie relationships between higher adult child educational attainment and psychosocial outcomes for older parents during the pandemic.

Beyond the economic realm, studies using pre-pandemic data have suggested that higher adult child educational attainment may contribute to older parents’ health care access and health behaviors, which may be relevant for pandemic-era outcomes. For example, studies have found that higher adult child educational attainment is associated with better management of chronic health conditions [29, 30] and a lower risk of adverse health behaviors (e.g. smoking) among older parents [20, 31]. These associations are often explained by the intergenerational transmission of knowledge and information about health risks as well as direct and indirect support in navigating the health care system (e.g. communicating with health care providers) [32, 33]. Specifically in the context of COVID-19, higher adult child SES could have yielded greater awareness of the risks associated with in-person contact for COVID-19 transmission amongst older parents. Research using SHARE data has shown the importance of close kin for the transmission of knowledge about COVID vaccination as well as instrumental support to help avoid the risk of infection [34]. Reduced risk of infection or reduced risk of adverse outcomes even in the presence of infection could have contributed to reduced worry and anxiety among older adults [35].

In the present study, we bridge recent literature on socio-economic inequalities in psychosocial outcomes among older adults during the COVID-19 pandemic with pre-pandemic literature on the “upward” intergenerational influences (i.e., the directional flow of influence from adult children to parents) of adult child SES on older parents’ health. We evaluated whether older European adults’ psychosocial outcomes during the COVID-19 pandemic were patterned by adult child educational attainment—a core maker of SES- independent of respondents’ own lifecourse SES. We expected that higher adult child educational attainment would be associated with better psychosocial outcomes among older parents during the pandemic. We additionally expected that higher adult child educational attainment would be associated with a lower risk of older parents perceiving that their psychosocial outcomes had worsened compared to the pre-pandemic period. We secondarily evaluated associations between adult child educational attainment and older parents’ frequency of contact with children, the prevalence of help with basic needs given to and received from children, and respondents’ COVID-19-specific outcomes. These latter outcomes could serve as potential pathways of influence between adult child educational attainment and parents’ psychosocial outcomes.

Finally, we evaluated whether associations between adult child educational attainment and older adults’ psychosocial outcomes varied across both time and pandemic stage. Specifically, we expected that adult child educational attainment could have been more impactful for parents’ outcomes during the earliest months of COVID-19, characterized by scarcity of resources to cope with the impacts of lockdown, including not only basic resources like food and toiletries, but also essential mental and health support. We also considered that there could be heterogeneity based on the intensity of COVID-19, given that European countries experienced the pandemic differently at any given point in time and country-level pandemic intensity has been independently associated with population mental health [1, 4, 5].

## Methods

### Data

Data for this study comes from the Survey of Health, Ageing and Retirement in Europe (SHARE) [36] and the SHARE Corona Survey (SCS) [37, 38]. SHARE has collected data on adults ≥ 50 years of age and their spouses since 2004 with approximately biennial follow-ups through early 2020; 28 European countries and Israel have been included in SHARE. The SCS was conducted among SHARE panel members in wave 8 via computer-assisted telephone interviews (CATI) during the early months of the pandemic (June–August 2020) and one year later (June–August 2021).

We restricted the analytic sample to respondents ≥ 65 years in European countries (excluding Israel); we focused on adults ≥ 65 given that older adults were disproportionately impacted by the COVID-19 pandemic in terms of both morbidity and mortality as well as some adverse psychosocial sequelae (e.g. social isolation) [39, 40]. We followed a complete case analysis approach and further restricted the analytic sample to those who: 1) had at least one living child, 2) reported on their child characteristics at any of the pre-pandemic SHARE waves, and 3) had completed SHARE wave 8 (conducted between October 2019 and March 2020), from which we derived our pre-pandemic control variables, and 4) had complete data in either one or both SCS waves. After applying these inclusion criteria, we were left with an analytic pooled sample of 40,392 respondents (see eFig. 1).

### Outcome measures

#### Psychosocial outcomes

At each of the two SCS waves, participants answered the following questions, which each had binary yes/no response options: *“In the last month, have you felt nervous, anxious, or on edge?”, “In the last month, have you been sad or depressed?”, “Have you had trouble sleeping recently?”.* Respondents also reported on the frequency of feeling lonely; we grouped those who reported often or some of the time (vs. hardly ever or never).

During the first SCS wave, respondents were asked to rate whether each of the symptoms listed above (feeling nervous, feeling sad or depressed, having sleep problems, and feeling lonely) had worsened, improved, or stayed the same as compared to the pre-pandemic period. We contrasted those who perceived that their symptoms had worsened with those who reported perceiving that their symptoms had improved, stayed the same, or that they had not experienced that symptom. Worsened psychosocial outcomes over time (e.g. increases in depressive symptoms, increases in loneliness) as well as poorer sleep over time have been associated with a greater risk of poor subsequent outcomes (e.g. stroke, dementia, mortality) [41–45].

#### Secondary outcomes

Contact with children was measured at each pandemic wave with the following question: *“Since the outbreak of Corona, how often did you have personal contact, that is, face to face with your own children from outside your home?”.* We grouped those who responded daily, several times a week, or about once a week (vs. less often or never).

Support given to and received from children was measured with two questions at each wave: *“How often did your own children help you to obtain necessities, compared to before the outbreak of Corona? Less often, about the same, or more often?”. “Compared to before the outbreak of Corona, how often did you help your own children to obtain necessities: less often, about the same, or more often?”* Wording for the latter two questions shifted slightly during the second SCS wave (see Supplemental Appendix for specific question wording). We grouped those who responded less often or about the same (vs. more often).

Respondents’ COVID-19 experiences were captured with the following questions at each wave, which we evaluated as separate outcomes: *“Have you or anyone close to you been tested for the Corona virus and the result was positive, meaning that the person had the Covid disease? (Yes/No)”, Have you or anyone close to you been hospitalized due to an infection from the Corona virus? (Yes/No)”, “Has anyone close to you died due to an infection from the Corona virus? (Yes/No)”.*

### Adult child educational attainment

At the pre-pandemic SHARE waves, respondents reported the level of educational attainment for each of their children ≥ 16 years of age. Levels of educational attainment were standardized across SHARE countries using the International Standard Classification of Education (ISCED-1997) [46], with values that ranged from 0 (pre-primary education) to 6 (doctoral studies). For each respondent, we calculated a binary variable contrasting those who reported that their adult children had completed a mean level of ISCED-1997 classification at or above vs. below the mean level for all respondents residing in the same country, with means calculated based on the entire SHARE survey (i.e. before applying exclusion and inclusion criteria).

To evaluate the consistency of our results in sensitivity analyses, we created 1) a continuous measure of the average ISCED-1997 score (range: 0–6) that was not standardized to country-specific averages, 2) a categorical variable based on country-specific quartiles of average adult child educational attainment, and 3) a binary indicator of mean levels of ISCED-1997 classification for adult children, comparing those at or above the mean level to those below the mean level for all respondents in the same country, based on baseline or first available ISCED-1997 classification values.

### Confounders and effect modifiers

We followed modern epidemiologic principles of confounder selection [47, 48] and considered confounders to be measures that may have influenced adult–child educational attainment – which was generally established long before the pandemic – and older parents’ psychosocial outcomes during the COVID-19 pandemic. These included respondent’s age, sex, educational attainment, nativity, marital status, parents’ level of educational attainment (for mother and father), spouse’s age (if currently married/partnered, with an indicator variable for whether or not respondents had a current spouse/partner to retain the entire analytic sample), spouse’s educational attainment (if currently married/partnered or formerly married/partnered, with an indicator variable for whether or not respondents had a current or former spouse/partner to retain the entire analytic sample), the total number of respondent’s living children, the percentage of female children, and country fixed effects. All these measures were captured during pre-pandemic waves of data collection. We did not control for respondents’ pre-pandemic health, given that this may have mediated the relationship between adult child educational attainment and psychosocial outcomes during the pandemic.

We considered variation in associations by SCS wave, which corresponded to some of the earliest months of the pandemic (Wave 1) and the post-vaccination period (Wave 2). We also considered heterogeneity by the intensity of the COVID-19 pandemic since higher country-level COVID intensity has been associated with population mental health [1, 4, 5]. COVID-19 intensity was measured as the number of cases per 1,000 population averaged separately for each country during the three-month data collection period for the first SCS wave; data were obtained via Our World in Data, sourced from Johns Hopkins University [49]. We transformed this measure into a binary indicator of cases per 1000 population at or above vs. below the median across the countries included in our analyses. We focused our analysis on heterogeneity by COVID-19 intensity on the first SCS wave (June – August 2020); given the vast differences in the landscape of vaccination and treatment by the second SCS wave (June – August 2021), the meaning of “high” COVID-19 intensity may have varied substantially by this time. We replicated the same procedure using the number of deaths per 1,000 population to assess the consistency of our results across measures of COVID intensity.

### Analytic strategy

We used generalized estimating equations (GEE) with a Poisson distribution and a log link [50] to analyze the associations between adult child educational attainment and older parents’psychosocial outcomes, contact with children, support given to and received from children, and COVID-19 experiences, adjusting for all confounders described above. Our models were estimated using robust standard errors. We pooled data across both pandemic waves, which allowed for improved precision. The GEE models account for the non-independence of repeated observations of the same respondents over the two pandemic waves.

We used inverse probability weights to account for selective missingness from the analytic sample, with missingess driven by respondents either not having children, having children but lacking data on the exposure variable, or having children but with missing values on outcome variables (Appendix Fig. 1). To create the weights, we estimated a logistic regression model in which our outcome was a binary indicator of inclusion (vs. exclusion) from our analytic sample, conditional on respondents’ demographic characteristics (i.e. age, sex, educational attainment, nativity, marital status, and country of origin). We generated predicted probabilities of inclusion from this model, which we used to calculate weights equal to the inverse of the probability of inclusion (i.e. 1/*p*_inclusion_) for those who were included in the analytic sample. We did not adjust or trim the weights in any way.

**Fig. 1:**
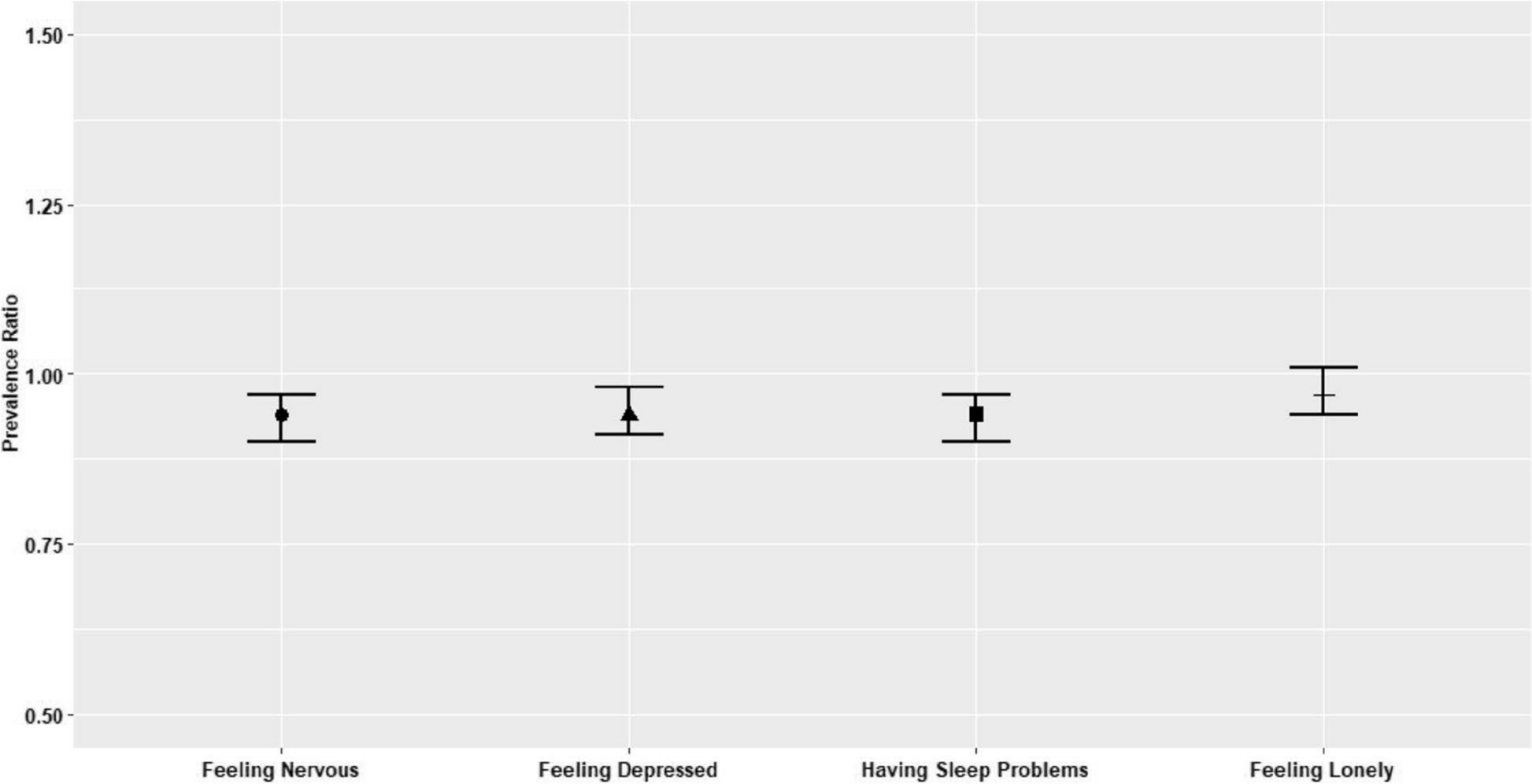
Adult Child Educational Attainment and Older Parents’ Psychosocial Outcomes During the COVID-19 Pandemic. Source: Pooled observations of respondents in the SHARE Corona Survey, Wave 1 and Wave 2, of the Survey on Health, Aging and Retirement in Europe (SHARE) (*N* = 40,392). Notes: Exposure is a binary indicator of mean levels of ISCED-1997 classification for adult children, comparing those at or above the mean level to those below the mean level for all respondents in the same country. Controls include age, sex, educational attainment, nativity, country, marital status, parental education (mother and father), age of current spouse (if married/partnered) and educational attainment of current or former spouse, number of children and percentage of female children

While our main models used pooled data from both pandemic waves to examine associations with our primary and secondary outcomes, we also conducted further analyses to examine whether the associations with our primary outcomes varied according to the sex/gender of the adult child and the respondent. This additional testing is motivated by prior evidence of heterogeneity in studies of adult child educational attainment and pre-pandemic health outcomes for older parents [14, 18, 20, 22]. We evaluated effect estimates and 95% confidence intervals in models that considered adult child education separately for daughters vs. sons. Additionally, we evaluated heterogeneity by parent sex/gender with stratified models, which allowed us to compare effect estimates and 95% confidence intervals.

We subsequently evaluated potential differences in associations with our primary outcomes across study waves and country-level COVID-19 intensity by comparing effect estimates and 95% confidence intervals in stratified models (i.e. for Wave 1 vs. Wave 2 and high vs. low COVID intensity measures) and formally tested multiplicative interaction terms in pooled models e.g. evaluating the multiplicative interaction between study wave and adult child educational attainment in association with parents’ outcomes.

In order to evaluate heterogeneity, we compared the sign and magnitude of effect estimates and the overlap in 95% confidence intervals across stratified models. When we additionally tested for interaction in pooled models, we also considered the effect estimates and *p*-values corresponding to multiplicative interaction terms.

Finally, we assessed the consistency of our findings from the main models which examined associations with our primary and secondary outcomes. This evaluation used both a continuous measure of the average ISCED-1997 score (range: 0–6) that was not standardized to country-specific averages as well as a categorical variable based on country-specific quartiles of average adult child educational attainment. Moreover, we confirmed the reliability of the outcomes from our main models related to primary outcomes through the application of baseline or first available ISCED-1997 classification values.

## Results

### Descriptive statistics

Respondents’ characteristics are summarized for the first and second waves of the SCS in Table 1. In each wave, respondents had a median age of 74 (Interquartile Range [IQR]: 69–80) and 73 (IQR: 69–79), respectively, they were primarily female (57% and 58%), born in their country of residency (93%), and married (68%). Respondents had a median ISCED level of 3 (IQR: 2–4), roughly corresponding to lower secondary educational attainment. Respondents had a median of two (IQR 2–3) adult children with a median ISCED level of 4 (IQR: 3–5), corresponding to an upper secondary level of education.

**Table 1:** Descriptive Characteristics, Adults 65 + Years in SHARE and SHARE Corona Survey Wave 1 and Wave 2

	Wave 1 (*N* = 20,916)	Wave 2 (*N* = 19,476)
**Pre-COVID Socio-Demographic Characteristics**		
Age, median (IQR)	74 (69–80)	73 (69–79)
Female, n (%)	11,959 (57.2)	11,267 (57.9)
Native-born, n (%)	19,409 (92.8)	18,057 (92.7)
Marital status, n (%)		
Married/partnered	14,198 (67.9)	13,332 (68.5)
Widowed	4,951 (23.7)	4,444 (22.8)
Divorced/separated	1,510 (7.2)	1,460 (7.5)
Single	257 (1.2)	240 (1.2)
Educational attainment (range: 0–6), median (IQR)^a^	3 (2–4)	3 (2–4)
**Pre-COVID Parental Characteristics**		
Average parental educational attainment, n (%)^a^		
Low (range: 0–3)	17,363 (83.0)	16,158 (83.0)
High (range: 4–6)	1,048 (5.0)	1,012 (5.2)
Parental level of education not available	2,505 (12.0)	2,306 (11.8)
**Pre-COVID Spouse Characteristics**		
Age of current spouse (if married/partnered), n (%)		
< 65	1,515 (7.2)	1,505 (7.7)
65–79	9,326 (44.6)	8,924 (45.8)
80 +	2,733 (13.1)	2,349 (12.1)
Age of spouse not available	7,342 (35.1)	6,698 (34.4)
Spousal educational attainment, n (%)		
Low (range: 0–3)	13,234 (63.3)	12,270 (63.0)
High (range: 4–6)	4,357 (20.8)	4,279 (22.0)
Spousal level of education not available	3,325 (15.9)	2,927 (15.0)
**Pre-COVID Child Characteristics**		
Total number of children, median (IQR)	2 (2–3)	2 (2–3)
Female children, n (%)	10,301 (49.2)	9,596 (49.3)
Average educational attainment (range: 0–6), median (IQR)^a^	4 (3–5)	4 (3–5)
**Outcomes and Experiences during COVID**		
** Covid Intensity**		
Covid cases per 1,000 population, median (IQR)	143 (101–307)	-
Covid deaths per 1,000 population, median (IQR)	5.1 (3.2–19.3)	-
**Psychosocial, n (%)**		
Feeling nervous	6,008 (28.7)	6,254 (32.1)
Feeling depressed	5,460 (26.1)	5,992 (30.8)
Having sleep problems	5,914 (28.3)	6,318 (32.4)
Feeling lonely	6,274 (30.0)	6,268 (32.2)
Worsened feelings of nervousness	4,252 (20.3)	-
Worsened feelings of depression	3,405 (16.3)	-
Worsened sleep problems	1,608 (7.7)	-
Worsened feelings of loneliness	2,631 (12.6)	-
**Family Relationships and Contact, n (%)**		
Little or no contact with children	9,785 (46.8)	5,947 (30.5)
Received help from child to obtain necessities since the outbreak	4,994 (66.5)	7,312 (37.9)
Helped child to obtain necessities since the outbreak	249 (18.4)	1,875 (9.7)
**Overall Covid Outcomes, n (%)**		
Anyone tested positive for COVID-19	1,269 (6.1)	6,954 (35.7)
Anyone hospitalized due to COVID-19	630 (3.0)	2,306 (11.8)
Anyone died due to COVID-19	497 (2.4)	1,580 (8.1)

Source: Survey on Health, Aging and Retirement in Europe

^a^Educational levels based on the International Standard Classification of Education (ISCED, 1997), with values that ranged from 0 (pre-primary education) to 6 (doctoral studies)

Across the two SCS waves (Wave 1 and Wave 2), 29% and 32% of respondents reported feeling nervous, 26% and 31% reported feeling sad or depressed (hereafter “feeling depressed” for brevity), 28% and 32% reported having sleep problems, and 30% and 32% reported feeling lonely. In the first wave, one-fifth of respondents perceived that their feelings of nervousness had worsened as compared to before the COVID-19 outbreak, 16% perceived that their feelings of depression had worsened, 8% reported worsened sleep problems, and 13% felt worsened feelings of loneliness.

In addition, across SCS Wave 1 and Wave 2, 47% and 30% of respondents reported little or no in-person contact with their children, 67% and 38% received help more often from children to obtain basic necessities, and 18% and 10% reported helping their children more often obtain basic necessities. Finally, 6% and 36% reported a positive COVID-19 test, 3% and 12% reported COVID-19-related hospitalization for themselves or someone close to them, and 2% and 8% reported that someone close to them had died of COVID-19.

### Associations with psychosocial outcomes during the COVID-19 pandemic

Respondents for whom average adult child educational attainment was at or above the country-specific mean had a 6% lower risk of reporting feeling nervous (95% Confidence Interval [CI]: 0.90, 0.97), a 6% lower risk of reporting feeling depressed (95% CI: 0.91, 0.98), a 6% lower risk of having sleep problems (95% CI: 0.90, 0.97) during the pandemic compared to respondents for whom average adult child educational attainment was below the country-specific mean (Fig. 1). Higher average adult child educational attainment was associated with a 3% lower risk of feeling lonely (95% CI: 0.94, 1.01), although the 95% CI crossed the null. Results were substantively similar when using the continuous and categorical measures of adult child educational attainment (Table 2). Similar substantial consistency was observed when using the mean levels of ISCED-1997 classification for adult children, comparing those at or above the mean level to those below the mean level for all respondents in the same country, based on baseline or first available ISCED-1997 classification values (Appendix Table 1).

**Table 2:** Adult Child Educational Attainment and Older Parents’ Psychosocial Outcomes During the COVID-19 Pandemic

	Feeling Nervous		Feeling Depressed		Having Sleep Problems		Feeling Lonely	
	PR	95% CI	PR	95% CI	PR	95% CI	PR	95% CI
High Adult Child Educational Attainment^a^	0.94***	0.90—0.97	0.94***	0.91—0.98	0.94***	0.90—0.97	0.97	0.94—1.01
Continuous	0.96***	0.94—0.97	0.96***	0.95—0.98	0.96***	0.94—0.98	0.99*	0.97—1.00
Quartiles								
1 (Reference category)								
2	0.95**	0.90—0.99	0.97	0.93—1.02	0.96	0.92—1.01	0.98	0.93—1.02
3	0.94***	0.90—0.98	0.95**	0.91—0.99	0.95**	0.91—0.99	0.98	0.94—1.02
4	0.89***	0.84—0.94	0.91***	0.86—0.97	0.91***	0.86—0.97	0.96	0.91—1.02

Source: Pooled observations of respondents in the SHARE Corona Survey, wave 1 and wave 2, of the Survey on Health, Aging and Retirement in Europe (SHARE) (*N* = 40,392).

^a^Exposure is a binary indicator of mean levels of ISCED-1997 classification for adult children, comparing those at or above the mean level to those below the mean level for all respondents in the same country. Controls include age, sex, educational attainment, nativity, country, marital status, parental education (mother and father), age of current spouse (if married/partnered) and educational attainment of current or former spouse, number of children and percentage of female children

^***^p < 0.001, ** *p* < 0.01, * *p* < 0.05

**Fig. 2:**
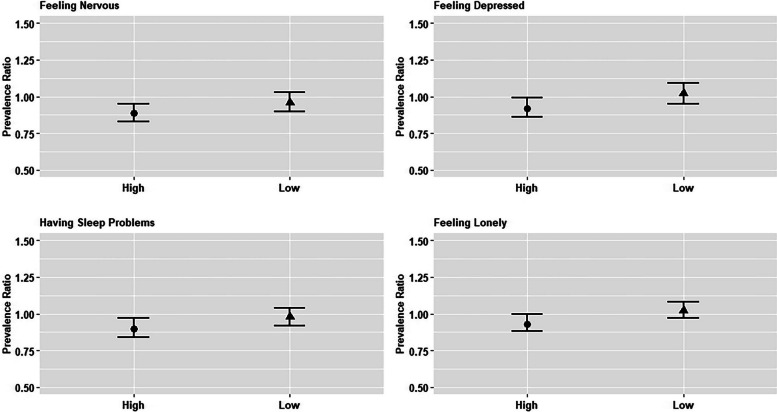
. Adult Child Educational Attainment and Older Parents’ Psychosocial Outcomes, by COVID-19 Intensity. Source: SHARE Corona Survey Wave 1 of the Survey on Health, Aging and Retirement in Europe (SHARE) (*N* = 10,340 for high-country-level COVID-19 intensity and *N* = 10,576 for low-country-level COVID-19 intensity). Notes: Exposure is a binary indicator of mean levels of ISCED-1997 classification for adult children, comparing those at or above the mean level to those below the mean level for all respondents in the same country. Controls include age, sex, educational attainment, nativity, country, marital status, parental education (mother and father), age of current spouse (if married/partnered) and educational attainment of current or former spouse, number of children and percentage of female children

There was no evidence of heterogeneous associations by SCS wave based on estimates presented in stratified models and *p*-values corresponding to multiplicative interaction terms in pooled models (Appendix Table 2). There was evidence of heterogeneity by COVID-19 intensity, measured as the number of cases per 1,000 population, based on both effect estimates in stratified models (Fig. 2) and the multiplicative interaction terms evaluated in pooled models (Appendix Table 3) for the outcomes of feeling nervous and feeling lonely. There was no evidence of heterogeneity in associations with feeling depressed or having sleep problems. Respondents in countries experiencing higher levels of COVID-19 intensity during the first SCS wave and with average adult child educational attainment at or above the country-specific mean had an 11% lower risk of reporting feeling nervous (95% CI: 0.83, 0.95), a 7% lower risk of feeling lonely (95% CI: 0.88, 1.00) compared to respondents for whom average adult child educational attainment was below the country-specific mean (Fig. 2). Conversely, there were no significant associations observed in countries with lower COVID-19 intensity (Fig. 2). Substantially similar outcomes were observed when using the number of deaths per 1,000 population as our COVID-19 intensity measure, instead of the number of cases per 1,000 population (Appendix Table 4).

There was no indication of heterogeneity by adult child sex/gender, as demonstrated by the overlapping 95% confidence intervals. However, when examining the educational attainment of adult daughters, we observed larger effect sizes: an 8% lower risk of reporting nervousness, a 5% lower risk of reporting depression, a 5% lower risk of experiencing sleep problems, and a 2% lower risk of feeling lonely (although the 95% CI for the outcome feeling lonely crossed the null). In contrast, for adult sons, the effect sizes were smaller: a 2% lower risk of reporting nervousness (which crossed the null), a 2% lower risk of reporting depression (also crossing the null), a 5% lower risk of experiencing sleep problems, and a 4% lower risk of feeling lonely (Appendix Table 5). While 95% confidence intervals overlapped for estimates stratified by parent sex, the magnitude of associations was larger for fathers (9% lower risk of reporting nervousness, 9% lower risk of reporting depression, 8% lower risk of experiencing sleep problems, and 9% lower risk of feeling lonely) compared to mothers (5% lower risk of reporting nervousness, 4% lower risk of reporting depression, 5% lower risk of experiencing sleep problems, and 0% lower risk of feeling lonely, although the 95% CI for the latter outcome crossed the null) (Appendix Table 6).

### Associations with perceptions of worsened psychosocial outcomes

**Fig. 3:**
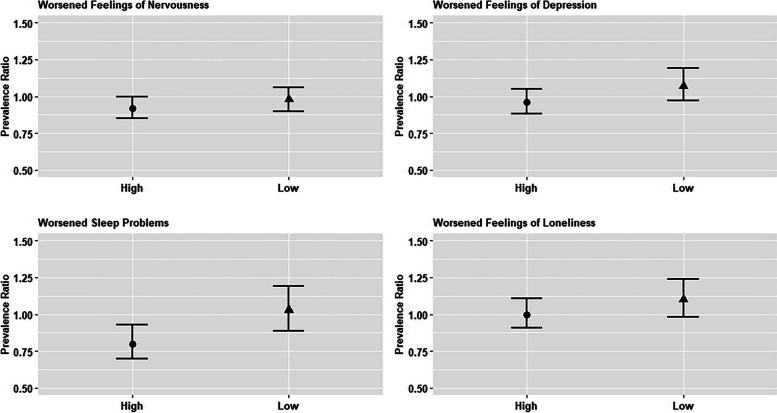
Adult Child Educational Attainment and Older Parents’ Psychosocial Outcomes Compared to the Pre-Pandemic Period, by COVID-19 Intensity. Source: SHARE Corona Survey Wave 1 of the Survey on Health, Aging and Retirement in Europe (SHARE) (*N* = 10,340 for high-country-level COVID-19 intensity and *N* = 10,576 for low-country-level COVID-19 intensity). Notes: Exposure is a binary indicator of mean levels of ISCED-1997 classification for adult children, comparing those at or above the mean level to those below the mean level for all respondents in the same country. Controls include age, sex, educational attainment, nativity, country, marital status, parental education (mother and father), age of current spouse (if married/partnered) and educational attainment of current or former spouse, number of children and percentage of female children

Respondents for whom average adult child educational attainment was at or above the country-specific mean had a 5% lower risk of perceiving worsened feelings of nervousness (95% CI: 0.90, 1.01) and a 9% lower risk of perceiving worsened sleep problems (95% CI: 0.82, 1.01), compared to those for whom average adult child educational attainment was below the country-specific mean. In contrast, there were no significant associations observed with the outcomes of perception of worsened feelings of depression and loneliness (Appendix Table 7). There was evidence of heterogeneity by country-level COVID intensity for perceived worsened feelings of nervousness and perceived worsened sleep problems. This evidence was observed through effect estimates in stratified models (Fig. 3) and the evaluation of multiplicative interaction terms in pooled models (Appendix Table 8). Respondents in countries experiencing higher levels of COVID-19 intensity during the first SCS wave and with average adult child educational attainment at or above the country-specific mean had an 8% lower risk of perceiving worsened feelings of nervousness (95% CI: 0.85, 1.00) compared to respondents for whom average adult child educational attainment was below the country-specific mean. Similarly, they also had a 20% lower risk of perceiving worsened sleep problems (95% CI: 0.70, 0.93) compared to respondents for whom average adult child educational attainment was below the country-specific mean. Associations in countries with low COVID-19 intensity were generally null. Findings remained substantially consistent when using the number of deaths per 1,000 population as our COVID-19 intensity measure (Appendix Table 9).

There was no evidence of heterogeneity by adult child sex, as indicated by the overlapping 95% confidence intervals (Appendix Table 10), however, there were differences in the magnitude of associations with certain outcomes. Specifically, the associations with perceiving worsened feelings of nervousness was larger for daughters compared to sons, while the association with perceiving worsened feelings of sadness or depression (hereafter worsened feelings of depression for brevity) was larger for sons compared to daughters and in the opposite direction of what was initially hypothesized. Associations with the other outcomes were null. While 95% confidence intervals overlapped throughout, associations between higher adult child educational attainment and perceiving worsened feelings of nervousness and perceiving worsened sleep problems were stronger for fathers compared to mothers. Conversely, associations with perceiving worsened feelings of loneliness were stronger for mothers compared to fathers and in the opposite direction of what was hypothesized (Appendix Table 11). Associations with perceiving worsened feelings of depression were null.

### Associations with potential family and COVID-related mechanisms

Average adult child educational attainment at or above the country-level mean was associated with a 7% higher risk of having little or no in-person contact with children (95% CI: 1.04, 1.10) compared to average adult child educational attainment below the country-level mean (Appendix Table 12). Associations with either receiving help from or giving help to children to obtain necessities were generally null.

Higher average adult child educational attainment was associated with a 10% higher risk of testing positive for COVID-19 or having someone close that tested positive (95% CI: 1.06, 1.15) compared to average adult child educational attainment below the country mean, but not with having been hospitalized, knowing someone close that had been hospitalized with COVID-19, or having someone close that died of COVID-19 (Appendix Table 13). All results were substantively similar when using binary, continuous, and categorical measures of adult child educational attainment (Appendix Tables 14 and 15).

## Discussion

In our research focusing on older European adults in a population-based study, we found that higher adult child educational attainment – a core marker of socio-economic status – was associated with a lower risk of poor psychosocial outcomes during the COVID-19 pandemic, including feeling nervous, feeling depressed, and having sleep problems. This study extends growing evidence based on pre-pandemic data demonstrating the importance of adult child socio-economic status for older parents’ health [14–17, 27], but is the first to focus on parents’ outcomes during the COVID-19 pandemic.

Additionally, we found that higher adult child educational attainment was associated with a lower risk of respondents reporting that their feelings of nervousness and sleep problems had worsened as compared to the pre-pandemic period. We also found strong associations between high adult child educational attainment and respondents’ risk of poor psychosocial outcomes, including perceptions of worsened feelings of nervousness and worsened sleep problems compared to the pre-pandemic period in countries experiencing a higher number of COVID-19 cases and deaths during the survey period (in models focused on the first SCS wave only). These findings could suggest that adult child socio-economic resources may be most important for older parents’ wellbeing during periods of societal and health-related duress. However, we did not find evidence of heterogeneity by SCS wave, which may suggest that the specific pandemic-related country-level circumstances may have been a more important modifier than a broad time frame.

Moreover, we found that higher adult child educational attainment was associated with *less* in-person contact with children across the pandemic waves and a *higher* risk of reporting COVID-19 infection among oneself and/or someone close (e.g. a family member or friend). These findings have little precedent in the pre-pandemic literature, although one prior study found evidence of an association between higher adult daughter educational attainment and more frequent child contact for older parents in South Korea [22]. Our findings may have been driven by pandemic-specific circumstances. For example, it may have been the case that higher SES adult children may have been less likely to co-reside with older parents, which would have limited opportunities for contact during a time of pandemic-related physical distancing. In addition, higher SES children may have been more likely to have received information about the risks of in-person contact for COVID-19 transmission among older parents. Recent research suggests that family members played a pivotal role in supporting COVID-related prevention behaviors among older adults in the SHARE survey [34]. However, this could also be an artifact of greater testing resources among higher SES families or higher risk in some occupations that require higher levels of education (e.g. medical professionals) during the early pandemic period. Future research may further pinpoint the mechanisms underlying these results.

There was evidence of differences in the magnitude of association for older mothers and fathers, although patterns varied by outcomes and confidence intervals overlapped. Prior (i.e. pre-pandemic) studies have been extremely mixed in this regard. For example, some studies have found greater health returns to adult child education for mothers [14, 19] while others have reported associations for fathers only [18], while others have reported no evidence of heterogeneity [25, 51]. Limited evidence of heterogeneity may point to the consistent importance of adult child educational attainment for older mother and fathers in this context. Finally, we found little systematic evidence of heterogeneity in associations by adult child sex. There is no consensus in the pre-pandemic literature on the topic of heterogeneous associations between the SES of adult daughters vs. sons and the outcomes of older parents; prior studies in the European context have found mixed evidence of heterogeneity in associations by child sex/gender, including evidence of no differences [18, 20, 25].

### Limitations

We acknowledge important study limitations, including residual confounding due to respondents’ long-standing economic and health conditions, including psychosocial wellbeing, that may have preceded the educational trajectories of their adult children and may have influenced both adult child educational attainment and pandemic outcomes. While respondents did report on health and economic circumstances in pre-COVID SHARE study waves, adult child educational attainment had generally been established for those in our analytic sample by their SHARE baseline wave, meaning that even respondents’ baseline health and economic measures would have likely been influenced by adult child educational attainment, and should not be included as confounders. Specifically: 1) only 9% of respondents who had the same number of adult children changed exposure status on the binary adult child educational attainment at some point across SHARE study waves, and 2) the estimated average of respondent’s average ages of adult children at their baseline waves was 38. Both points (1 and 2) reflect the fact that adult child educational attainment was largely ‘set’ by study baseline. The ideal study design would collect such data from mid-life adults as their children are completing their educational trajectories to better account for confounding due to parents’ characteristics.

In addition, there is no consensus on the best metric of adult child educational attainment in the presence of multiple children. We have focused on average attainment, but welcome further research that may explore further differences across the many possible specifications of adult child educational attainment in families with more than one child, such as the maximum, the minimum, the attainment of the oldest child, the youngest child, or the difference in schooling between children.

While we offered two ways of measuring COVID intensity, we acknowledge that there are multiple alternative metrics, including those that reflect within-country variation or those modeled as linear or non-linear terms; other relevant measures might be related to government-level pandemic policies (e.g. related to physical distancing) or economic policies [1, 4, 5]. Our intention was not to evaluate a comprehensive set of pandemic-related measures in our analyses, but rather to offer up a set of tests to see whether our main associations of interest (i.e. between adult child educational attainment and pandemic outcomes) were to some extent context-dependent.

Additionally, there may be measurement error in exposure and outcome variables. We note that measurement error in parents’ reports of the educational attainment of their adult children could be differential with respect to correlates of parental mental wellbeing (e.g. cognitive impairment). Furthermore, while educational attainment may be an important driver of contemporaneous SES, more proximate dimensions of adult child SES – including current income and wealth [52] – were not available. We also note we could not evaluate heterogeneity by adult child co-residence or consider whether patterns might be explained by co-residence dynamics [53], since the co-residence status of specific adult children was not evaluated during the pandemic waves. Finally, we note that these findings may not be generalizable across global settings with distinct social, economic, and health-related resources available during the pandemic, different average levels of intergenerational educational attainment, and distinct patterns of resource transmission between parents and their adult children.

## Conclusion

In this population-based study of older European adults, adult child educational attainment measured before the pandemic was associated with older parents’ psychosocial outcomes during the pandemic. Associations between adult child educational attainment and parents’ psychosocial outcomes and perceptions of worsened psychosocial outcomes (vs. the pre-pandemic period) were found in countries experiencing higher COVID-19. Higher adult child educational attainment was also associated with other parental outcomes during the pandemic, albeit in unexpected ways: a lower risk of in-person contact with adult children and a higher risk of experience with COVID-19 infection. This is the first study, to our knowledge, to empirically consider the role of adult child educational attainment in shaping older parents’ outcomes during the COVID-19 pandemic. Our findings suggest that adult child educational attainment is associated with the psychosocial wellbeing of older parents, particularly during periods of societal duress.

### Supplementary Information

Supplementary Material 1.

Supplementary Material 2.

## Data Availability

The datasets used in this study are publicly available through the official website of The Survey of Health, Ageing, and Retirement in Europe (SHARE): https://share-eric.eu/data/data-access. We can provide our analyses code upon request to facilitate replication of our findings.

## References

[1] Mendez-Lopez A, Stuckler D, McKee M, Semenza JC, Lazarus JV. The mental health crisis during the COVID-19 pandemic in older adults and the role of physical distancing interventions and social protection measures in 26 European countries. SSM Popul Health. 2022;17:101017. 10.1016/j.ssmph.2021.101017. 34977323 10.1016/j.ssmph.2021.101017 PMC8713431

[2] Richter L, Heidinger T. Hitting close to home: the effect of COVID-19 illness in the social environment on psychological burden in older adults. Front Psychol. 2021;12:737787. 10.3389/fpsyg.2021.737787. 10.3389/fpsyg.2021.737787 PMC8502856 34646219

[3] Koma W, True S, Fuglesten Biniek J, Juliette Cubanski J, Orgera K, Garfield R. One in four older adults report anxiety or depression amid the COVID-19 pandemic. KFF; 2020. https://www.kff.org/medicare/issue-brief/one-in-four-older-adults-report-anxiety-or-depression-amid-the-covid-19-pandemic/. Accessed July 2024.

[4] Bu F, Steptoe A, Fancourt D. Depressive and anxiety symptoms in adults during the COVID-19 pandemic in England: a panel data analysis over 2 years. PLoS Med. 2023;20(4):e1004144. 10.1371/journal.pmed.1004144. 10.1371/journal.pmed.1004144 PMC10112796 37071605

[5] Aknin LB, Andretti B, Goldszmidt R, et al. Policy stringency and mental health during the COVID-19 pandemic: a longitudinal analysis of data from 15 countries. Lancet Public Health. 2022;7(5):e417–26. 10.1016/S2468-2667(22)00060-3. 35461592 10.1016/S2468-2667(22)00060-3 PMC9023007

[6] Zaninotto P, Iob E, Demakakos P, Steptoe A. Immediate and longer-term changes in the mental health and well-being of older adults in England during the COVID-19 pandemic. JAMA Psychiat. 2022;79(2):151–9. 10.1001/jamapsychiatry.2021.3749. 10.1001/jamapsychiatry.2021.3749 PMC8696687 34935862

[7] Collinge AN, Bath PA. Socioeconomic background and self-reported sleep quality in older adults during the COVID-19 pandemic: an analysis of the English Longitudinal Study of Ageing (ELSA). Int J Environ Res Public Health. 2023;20(5):5–12. 10.3390/ijerph20054534. 10.3390/ijerph20054534 PMC10001974 36901540

[8] Samuel LJ, Dwivedi P, Hladek M, et al. The effect of COVID-19 pandemic-related financial challenges on mental health and well-being among US older adults. J Am Geriatr Soc. 2022;70(6):1629–41. 10.1111/jgs.17808. 35393645 10.1111/jgs.17808 PMC9115091

[9] O’Shea BQ, Finlay JM, Kler J, Joseph CA, Kobayashi LC. Loneliness among US Adults aged ≥55 early in the COVID-19 pandemic: findings from the COVID-19 coping study. Public Health Rep. 2021;136(6):754–64. 10.1177/00333549211029965. 34283657 10.1177/00333549211029965 PMC8579390

[10] Webb LM, Chen CY. The COVID-19 pandemic's impact on older adults' mental health: contributing factors, coping strategies, and opportunities for improvement. Int J Geriatr Psychiatry. 2022;37(1):1–3. 10.1002/gps.5647. 10.1002/gps.5647 PMC8646312 34729802

[11] Liu Y, Zhang Y, Chatterjee S. Financial hardship and depression experienced by pre-retirees during the COVID-19 pandemic: the mitigating role of stimulus payments. Appl Econ Lett. 2023;30(3):391–6. 10.1080/13504851.2021.1989364. 10.1080/13504851.2021.1989364

[12] Yahirun JJ, Sheehan CM, Mossakowski KN. Depression in later life: the role of adult children’s college education for older parents’ mental health in the United States. J Gerontol B Psychol Sci Soc Sci. 2020;75(2):389–402. 10.1093/geronb/gby135. 30412237 10.1093/geronb/gby135 PMC7530494

[13] Yahirun JJ, Sheehan CM, Hayward MD. Adult children’s education and changes to parents’ physical health in Mexico. Soc Sci Med. 2017;181:93–101. 10.1016/j.socscimed.2017.03.034. 28384483 10.1016/j.socscimed.2017.03.034 PMC5600815

[14] Ma M, Yahirun JJ, Sáenz J, Sheehan CM. Offspring educational attainment and older parents’ cognition in Mexico. Demography. 2021;58(1):75–109. 33612872 10.1215/00703370-8931725 PMC7894606

[15] De Neve JW, Harling G. Offspring schooling associated with increased parental survival in rural KwaZulu-Natal, South Africa. Soc Sci Med. 2017;176:149–57. 10.1016/j.socscimed.2017.01.015. 28153751 10.1016/j.socscimed.2017.01.015 PMC5322823

[16] De Neve JW, Kawachi I. Spillovers between siblings and from offspring to parents are understudied: a review and future directions for research. Soc Sci Med. 2017;06(183):56–61. 10.1016/j.socscimed.2017.04.010. 10.1016/j.socscimed.2017.04.010 28478353

[17] Thoma B, Sudharsanan N, Karlsson O, Joe W, Subramanian SV, De Neve JW. Children's education and parental old-age health: evidence from a population-based, nationally representative study in India. Popul Stud (Camb). 2020:1–16. 10.1080/00324728.2020.1775873. 10.1080/00324728.2020.1775873 32672098

[18] Lundborg P, Majlesi K. Intergenerational transmission of human capital: is it a one-way street? J Health Econ. 2018;01(57):206–20. 10.1016/j.jhealeco.2017.12.001. 10.1016/j.jhealeco.2017.12.001 29289810

[19] Torres JM, Yahirun JJ, Sheehan C, Ma M, Sáenz J. Adult child socio-economic status disadvantage and cognitive decline among older parents in Mexico. Soc Sci Med. 2021;279:113910. 10.1016/j.socscimed.2021.113910. 33964589 10.1016/j.socscimed.2021.113910 PMC8284312

[20] Torres JM, Yang Y, Rudolph KE, Courtin E. Increased adult child schooling and older parents’ health behaviors in Europe: a quasi-experimental study. SSM Popul Health. 2022;19:101162. 10.1016/j.ssmph.2022.101162. 35855968 10.1016/j.ssmph.2022.101162 PMC9287559

[21] Lee C. Adult children’s education and physiological dysregulation among older parents. J Gerontol B Psychol Sci Soc Sci. 2018;73(6):1143–54. 10.1093/geronb/gbx044. 28444349 10.1093/geronb/gbx044 PMC6093314

[22] Lee Y. Adult children’s educational attainment and the cognitive trajectories of older parents in South Korea. Soc Sci Med. 2018;209:76–85. 10.1016/j.socscimed.2018.05.026. 29803071 10.1016/j.socscimed.2018.05.026

[23] Yahirun JJ, Sheehan CM, Mossakowski KN. Depression in later life: the role of adult children’s college education for older parents’ mental health in the United States. J Gerontol B Psychol Sci Soc Sci. 2018. 10.1093/geronb/gby135. 30412237 10.1093/geronb/gby135 PMC7530494

[24] Gutierrez S, Courtin E, Glymour MM, Torres JM. Does schooling attained by adult children affect parents’ psychosocial well-being in later life? Using Mexico’s 1993 compulsory schooling law as a quasi-experiment. SSM Popul Health. 2024;25:101616. 10.1016/j.ssmph.2024.101616. 38434444 10.1016/j.ssmph.2024.101616 PMC10905038

[25] Torres JM, Yang Y, Rudolph KE, Meza E, Glymour MM, Courtin E. Adult child schooling and older parents’ cognitive outcomes in the Survey of Health, Aging and Retirement in Europe (SHARE): a quasi-experimental study. Am J Epidemiol. 2022;191(11):1906–16. 10.1093/aje/kwac151. 36040294 10.1093/aje/kwac151 PMC9767648

[26] Gilligan M, Suitor JJ, Rurka M, Silverstein M. Multigenerational social support in the face of the COVID-19 pandemic. J Fam Theory Rev. 2020;12(4):431–47. 10.1111/jftr.12397. 34367339 10.1111/jftr.12397 PMC8340915

[27] Ma M. Does children’s education matter for parents’ health and cognition? Evidence from China. J Health Econ. 2019;66:222–40. 10.1016/j.jhealeco.2019.06.004. 31265950 10.1016/j.jhealeco.2019.06.004

[28] Fingerman KL, Cheng YP, Birditt K, Zarit S. Only as happy as the least happy child: multiple grown children’s problems and successes and middle-aged parents’ well-being. J Gerontol B Psychol Sci Soc Sci. 2012;67(2):184–93. 10.1093/geronb/gbr086. 21856677 10.1093/geronb/gbr086 PMC3410695

[29] Larsen EN, Brunnich Sloth MM, Nielsen J, Osler M, Hoj Jorgensen TS. The association of children and their educational attainment with diabetes-related complications and mortality among older adults with type 2 diabetes: a nationwide cohort study. Can J Diabetes. 2023;47(8):649–57. 10.1016/j.jcjd.2023.07.004. 10.1016/j.jcjd.2023.07.004 37460085

[30] De León W, McLaughlin S. Adult children’s education and parents’ diabetes self-care behaviors in Mexico. Innov Aging. 2018;2(1):271. 10.1093/geroni/igy023.1003

[31] Friedman EM, Mare RD. The schooling of offspring and the survival of parents. Demography. A 2014;51(4):1271–93. 10.1007/s13524-014-0303-z. 24917296 10.1007/s13524-014-0303-z

[32] Ram A, Dave SS, Lancki N, et al. Social influence of adult children on parental health behavior among South Asian immigrants: findings from the MASALA (Mediators of Atherosclerosis in South Asians Living in America) study. Ethn Health. 2022;27(3):639–57. 10.1080/13557858.2020.1734779. 32122159 10.1080/13557858.2020.1734779 PMC8040023

[33] Jiang N, Kaushal N. How children’s education affects caregiving: evidence from parent’s last years of life. Econ Hum Biol. A 2020;38:100875. 10.1016/j.ehb.2020.100875. 32445917 10.1016/j.ehb.2020.100875

[34] Arpino B, Bordone V, Di Gessa G. COVID-19 precautionary behaviors and vaccine acceptance among older individuals: the role of close kin. Proc Natl Acad Sci U S A. 2023;120(13):e2214382120. 10.1073/pnas.2214382120. 36940329 10.1073/pnas.2214382120 PMC10068797

[35] Iob E, Steptoe A, Zaninotto P. Mental health, financial, and social outcomes among older adults with probable COVID-19 infection: a longitudinal cohort study. Proc Natl Acad Sci U S A. 2022;119(27):e2200816119. 10.1073/pnas.2200816119. 35763577 10.1073/pnas.2200816119 PMC9271189

[36] Börsch-Supan A, Brandt M, Hunkler C, et al. Data resource profile: the Survey of Health, Ageing and Retirement in Europe (SHARE). Int J Epidemiol. 2013;42(4):992–1001. 10.1093/ije/dyt088. 23778574 10.1093/ije/dyt088 PMC3780997

[37] Börsch-Supan A. Data from: Survey of Health, Ageing and Retirement in Europe (SHARE) wave 8. COVID-19 survey 1. 2022. Release version: 8.0.0

[38] Börsch-Supan A. Data from: Survey of Health, Ageing and Retirement in Europe (SHARE) Wave 9. COVID-19 Survey 2. 2022;Release version: 8.0.0.

[39] Sasson I. Age and COVID-19 mortality: a comparison of Gompertz doubling time across countries and causes of death. Demogr Res. 2021;44(16):379–96. 10.4054/DemRes.2021.44.16

[40] Williams RD II, Shah A, Doty MM, Fields K, FitzGerald M. The Impact of COVID-19 on older adults: findings from the 2021 International Health Policy Survey of Older Adults. New York: Commonwealth Fund; 2021. https://www.commonwealthfund.org/publications/surveys/2021/sep/impact-covid-19-older-adults. Accessed July 2024.

[41] Soh Y, Tiemeier H, Kawachi I, Berkman LF, Kubzansky LD. Eight-year depressive symptom trajectories and incident stroke: a 10-year follow-up of the HRS (Health and Retirement Study). Stroke. 2022;53(8):2569–76. 10.1161/STROKEAHA.121.037768. 35603598 10.1161/STROKEAHA.121.037768

[42] Saeed Mirza S, Ikram MA, Freak-Poli R, Hofman A, Rizopoulos D, Tiemeier H. 12-year trajectories of depressive symptoms in community-dwelling older adults and the subsequent risk of death over 13 years. J Gerontol A Biol Sci Med Sci. 2018;73(6):820–7. 10.1093/gerona/glx215. 29099907 10.1093/gerona/glx215

[43] Mirza SS, Wolters FJ, Swanson SA, et al. 10-year trajectories of depressive symptoms and risk of dementia: a population-based study. Lancet Psychiatry. 2016;3(7):628–35. 10.1016/S2215-0366(16)00097-3. 27138970 10.1016/S2215-0366(16)00097-3

[44] Wang YH, Wang J, Chen SH, et al. Association of longitudinal patterns of habitual sleep duration with risk of cardiovascular events and all-cause mortality. JAMA Netw Open. 2020;3(5):e205246. 10.1001/jamanetworkopen.2020.5246. 32442289 10.1001/jamanetworkopen.2020.5246 PMC7244989

[45] Li Y, Wang X, Guo L, et al. Eight-year trajectories of late-life loneliness and incident dementia: a nationally representative cohort study. Am J Geriatr Psychiatry. 2023;31(7):475–86. 10.1016/j.jagp.2022.12.002. 36549995 10.1016/j.jagp.2022.12.002

[46] UNESCO. International Standard Classification of Education: ISCED 1997, vol. 5. 2006. http://uis.unesco.org/sites/default/files/documents/international-standard-classification-of-education-1997-en_0.pdf.

[47] VanderWeele TJ. Principles of confounder selection. Eur J Epidemiol. 2019;34(3):211–9. 10.1007/s10654-019-00494-6. 30840181 10.1007/s10654-019-00494-6 PMC6447501

[48] Vansteelandt S, Bekaert M, Claeskens G. On model selection and model misspecification in causal inference. Stat Methods Med Res. 2012;21(1):7–30. 10.1177/0962280210387717. 21075803 10.1177/0962280210387717

[49] Dong E, Du H, Gardner L. An interactive web-based dashboard to track COVID-19 in real time. Lancet Infect Dis. 2020;20(5):533–4. 10.1016/S1473-3099(20)30120-1. 32087114 10.1016/S1473-3099(20)30120-1 PMC7159018

[50] Zou G. A modified poisson regression approach to prospective studies with binary data. Am J Epidemiol. 2004;159(7):702–6. 10.1093/aje/kwh090. 15033648 10.1093/aje/kwh090

[51] Yahirun JJ, Vasireddy S, Hayward MD. The education of multiple family members and the life course pathways to cognitive impairment. J Gerontol B Psychol Sci Soc Sci. 2020;75(7):e113–28. 10.1093/geronb/gbaa039. 10.1093/geronb/gbaa039 PMC7424275 32215643

[52] Torssander J. Adult children’s socioeconomic positions and their parents’ mortality: a comparison of education, occupational class, and income. Soc Sci Med. 2014;122:148–56. 10.1016/j.socscimed.2014.10.043. 25441327 10.1016/j.socscimed.2014.10.043

[53] Caputo J. Crowded nests: Parent-adult child coresidence transitions and parental mental health following the great recession. J Health Soc Behav. 2019;60(2):204–21. 10.1177/0022146519849113. 31122076 10.1177/0022146519849113 PMC6573002

