# Supplementary material for: Adult child educational attainment and older parents’ psychosocial outcomes during the COVID-19 pandemic
Source: BMC Public Health. 2024 Jul 31;24:2056. doi: 10.1186/s12889-024-19425-6 (PMC11289967; doi:10.1186/s12889-024-19425-6)
Supplement: Supplementary file 1 — Supplementary Material 1. [file 12889_2024_19425_MOESM1_ESM.docx]

**Adult child educational attainment and older parents’ psychosocial outcomes during the COVID-19 pandemic**

K. Renata Flores Romero,^a^ Yulin Yang,^a^ Sharon H. Green, ^b^ Sirena Gutierrez, ^a^ Erika Meza, ^a^ Jacqueline M. Torres ^a^

a. Department of Epidemiology & Biostatistics, UC San Francisco, San Francisco, CA

b. Department of Demography, UC Berkeley, Berkeley, CA

Corresponding Author:

Jacqueline M. Torres

550 16^th^ Street

San Francisco, CA 94143

Email: [Jacqueline.Torres@ucsf.edu](mailto:Jacqueline.Torres@ucsf.edu)

Phone: 415.317.3261

**Appendix**

**eFig. 1 Analytic Sample**


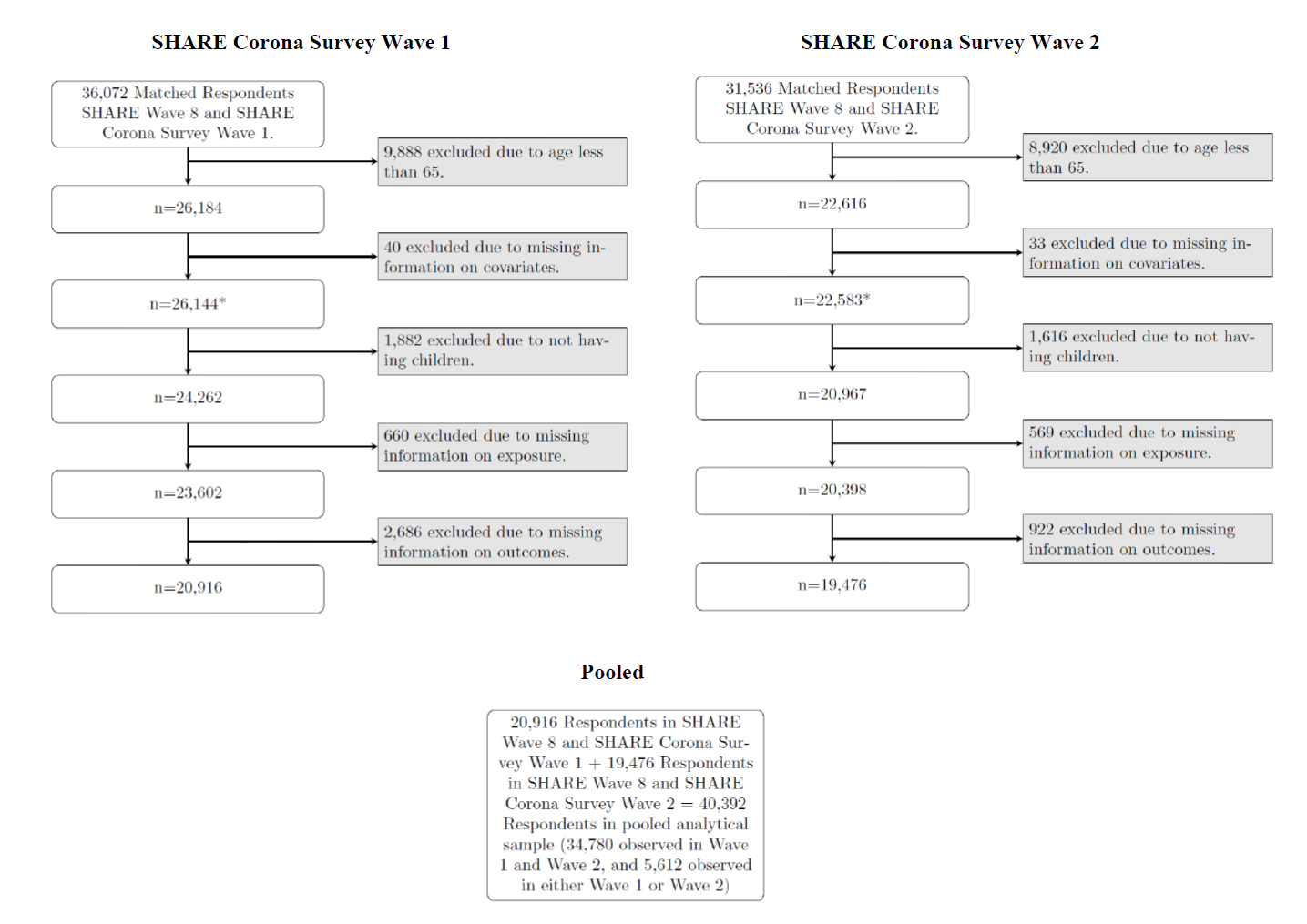


Notes: We use this sample to calculate inverse probability weights to account for missingness from the analytic sample stemming from the following reasons: 1) not having children, 2) having children but lacking data on the exposure variable, and 3) having children but with missing values on outcome variables.

| **eTable 1. Adult Child Educational Attainment and Older Parents' Psychosocial Outcomes During the COVID-19 Pandemic** | | | | | | | | |
| --- | --- | --- | --- | --- | --- | --- | --- | --- |
|  |  |  |  |  |  |  |  |  |
|  | Feeling Nervous | | Feeling Depressed | | Having Sleep Problems | | Feeling Lonely | |
|  | PR | 95% CI | PR | 95% CI | PR | 95% CI | PR | 95% CI |
| High Adult Child Educational Attainment | 0.96** | 0.93 - 1.00 | 0.96** | 0.92 - 0.99 | 0.96** | 0.93 - 1.00 | 0.98 | 0.95 - 1.02 |
| Continuous | 0.98** | 0.96 - 1.00 | 0.98** | 0.96 - 1.00 | 0.98** | 0.96 - 1.00 | 0.99 | 0.98 - 1.01 |
| Quartiles |  |  |  |  |  |  |  |  |
| 1 (Reference category) |  |  |  |  |  |  |  |  |
| 2 | 0.97 | 0.92 - 1.01 | 0.97 | 0.92 - 1.02 | 0.99 | 0.94 - 1.03 | 0.98 | 0.93 - 1.02 |
| 3 | 0.96* | 0.92 - 1.01 | 0.97 | 0.92 - 1.01 | 0.97 | 0.93 - 1.02 | 0.98 | 0.94 - 1.02 |
| 4 | 0.95* | 0.89 - 1.00 | 0.94* | 0.89 - 1.00 | 0.95* | 0.89 - 1.00 | 0.97 | 0.92 - 1.03 |
| Source: Pooled observations of respondents in the SHARE Corona Survey, wave 1 and wave 2, of the Survey on Health, Aging and Retirement in Europe (SHARE) (N=40,392). Notes: Exposure is a binary indicator of mean levels of ISCED-1997 classification for adult children, comparing those at or above the mean level to those below the mean level for all respondents in the same country, based on baseline or first available ISCED-1997 classification values. Controls include age, sex, educational attainment, nativity, country, marital status, parental education (mother and father), age of current spouse (if married/partnered) and educational attainment of current or former spouse, number of children and percentage of female children. *** *p* < 0.001, ** *p* < 0.01, * *p* < 0.05 | | | | | | | | |
|  |  |  |  |  |  |  |  |  |

| **eTable 2. Adult Child Educational Attainment and Older Parents' Psychosocial Outcomes During the COVID-19 Pandemic, by Wave** | | | | | | | | |
| --- | --- | --- | --- | --- | --- | --- | --- | --- |
|  |  |  |  |  |  |  |  |  |
|  | Feeling Nervous | | Feeling Depressed | | Sleep Problems | | Feeling Lonely | |
|  | PR | 95% CI | PR | 95% CI | PR | 95% CI | PR | 95% CI |
| **Wave 1** | | | | | | | | |
| High Adult Child Educational Attainment^a^ | 0.93*** | 0.89 - 0.97 | 0.97 | 0.92 - 1.02 | 0.95** | 0.90 - 0.99 | 0.98 | 0.94 - 1.03 |
| **Wave 2** | | | | | | | | |
| High Adult Child Educational Attainment^b^ | 0.94** | 0.90 - 0.99 | 0.92*** | 0.88 - 0.97 | 0.93*** | 0.89 - 0.97 | 0.96* | 0.92 - 1.00 |
| **Pooled** | | | | | | | | |
| High Adult Child Educational Attainment^c^ | 0.94*** | 0.90 - 0.98 | 0.96* | 0.91 - 1.00 | 0.93*** | 0.89 - 0.97 | 0.98 | 0.94 - 1.02 |
| High Adult Child Educational Attainment*  Study Wave 2 | 1.00 ^a^ | 0.95 - 1.05 | 0.97 ^b^ | 0.92 - 1.02 | 1.02 ^c^ | 0.97 - 1.07 | 0.98^d^ | 0.94 - 1.02 |
| Source: ^a^Respondents in SHARE Corona Survey Wave1 (N=20,916). ^b^Respondents in SHARE Corona Survey Wave1 (N=19,476). ^c^Pooled observations of respondents in the SHARE Corona Survey, wave 1 and wave 2, of the Survey on Health, Aging and Retirement in Europe (SHARE) (N=40,392). Notes: Exposure is the multiplicative interaction between a binary indicator of mean levels of ISCED-1997 classification for adult children, comparing those at or above the mean level to those below the mean level for all respondents in the same country, and a binary indicator contrasting wave 2 to wave 1. Controls include age, sex, educational attainment, nativity, country, marital status, parental education (mother and father), age of current spouse (if married/partnered) and educational attainment of current or former spouse, number of children and percentage of female children. ^a.^p-value= 0.850, ^b.^p-value= 0.212, ^c.^p-value= 0.464, ^d.^p-value= 0.341.*** *p* < 0.001, ** *p* < 0.01, * *p* < 0.05 | | | | | | | | |
|  |  |  |  |  |  |  |  |  |

| **eTable 3. Adult Child Educational Attainment and Older Parents' Psychosocial Outcomes During the COVID-19 Pandemic, by Country-Level COVID-19 Intensity (Cases)** | | | | | | | | |
| --- | --- | --- | --- | --- | --- | --- | --- | --- |
|  |  |  |  |  |  |  |  |  |
|  | Feeling Nervous | | Feeling Depressed | | Sleep Problems | | Feeling Lonely | |
|  | PR | 95% CI | PR | 95% CI | PR | 95% CI | PR | 95% CI |
| High Adult Child Educational Attainment | 0.96 | 0.91 - 1.03 | 1.00 | 0.93 - 1.07 | 0.97 | 0.92 - 1.03 | 1.02 | 0.97 - 1.08 |
| High Adult Child Educational Attainment*  High Covid-19 Intensity | 0.92^a^ | 0.85 - 1.01 | 0.95 ^b^ | 0.86 - 1.04 | 0.93 ^c^ | 0.85 - 1.02 | 0.92^d^ | 0.85 - 0.99 |
| Source: Respondents in the SHARE Corona Survey wave 1 of the Survey on Health, Aging and Retirement in Europe (SHARE) (N=20,916). Notes: Exposure is the multiplicative interaction between a binary indicator of mean levels of ISCED-1997 classification for adult children, comparing those at or above the mean level to those below the mean level for all respondents in the same country, and a country-level COVID-19 intensity binary indicator of cases/1000 population contrasting countries at or above the median to those below the median of all included countries in the SCS. Data for the country-level COVID-19 intensity indicator comes from Our World in Data, sourced from Johns Hopkins University. Controls include age, sex, educational attainment, nativity, country, marital status, parental education (mother and father), age of current spouse (if married/partnered) and educational attainment of current or former spouse, number of children and percentage of female children. ^a.^ p-value= 0.071, ^b.^ p-value= 0.234, ^c.^ p-value= 0.123, ^d.^ p-value= 0.037. *** *p* < 0.001, ** *p* < 0.01, * *p* < 0.05 | | | | | | | | |
|  |  |  |  |  |  |  |  |  |

| **eTable 4. Adult Child Educational Attainment and Older Parents' Psychosocial Outcomes During the COVID-19 Pandemic, by Country-Level COVID-19 Intensity (Deaths)** | | | | | | | | |
| --- | --- | --- | --- | --- | --- | --- | --- | --- |
|  |  |  |  |  |  |  |  |  |
|  | Feeling Nervous | | Feeling Depressed | | Sleep Problems | | Feeling Lonely | |
|  | PR | 95% CI | PR | 95% CI | PR | 95% CI | PR | 95% CI |
| High Adult Child Educational Attainment | 0.96 | 0.91 - 1.02 | 1.00 | 0.94 - 1.07 | 0.97 | 0.92 - 1.03 | 1.02 | 0.96 - 1.07 |
| High Adult Child Educational Attainment*  High Covid-19 Intensity | 0.93^a^ | 0.85 - 1.01 | 0.93^b^ | 0.85 - 1.02 | 0.93^c^ | 0.86 - 1.02 | 0.93^d^ | 0.85 - 1.00 |
| High Country-Level Covid-19 Intensity | | | | | | | | |
| High Adult Child Educational Attainment | 0.89*** | 0.83 - 0.95 | 0.92** | 0.85 - 0.98 | 0.91** | 0.85 - 0.98 | 0.94* | 0.88 - 1.01 |
| Low Country-Level Covid-19 Intensity | | | | | | | | |
| High Adult Child Educational Attainment | 0.96 | 0.90 - 1.02 | 1.02 | 0.95 - 1.10 | 0.97 | 0.92 - 1.04 | 1.02 | 0.96 - 1.07 |
| Source: Respondents in the SHARE Corona Survey wave 1 of the Survey on Health, Aging and Retirement in Europe (SHARE) (N=20,916). Notes: Exposure is the multiplicative interaction between a binary indicator of mean levels of ISCED-1997 classification for adult children, comparing those at or above the mean level to those below the mean level for all respondents in the same country, and a country-level COVID-19 intensity binary indicator of deaths/1000 population contrasting countries at or above the median to those below the median of all included countries in the SCS. Data for the country-level COVID-19 intensity indicator comes from Our World in Data, sourced from Johns Hopkins University. Controls include age, sex, educational attainment, nativity, country, marital status, parental education (mother and father), age of current spouse (if married/partnered) and educational attainment of current or former spouse, number of children and percentage of female children. ^a.^ p-value= 0.080, ^b.^ p-value= 0.132, ^c.^ p-value= 0.130, ^d.^ p-value= 0.061. *** *p* < 0.001, ** *p* < 0.01, * *p* < 0.05 | | | | | | | | |
|  |  |  |  |  |  |  |  |  |

| **eTable 5. Adult Child Educational Attainment and Older Parents' Psychosocial Outcomes During the COVID-19 Pandemic, by Adult Child Sex** | | | | | | | | |
| --- | --- | --- | --- | --- | --- | --- | --- | --- |
|  |  |  |  |  |  |  |  |  |
|  | Feeling Nervous | | Feeling Depressed | | Sleep Problems | | Feeling Lonely | |
|  | PR | 95% CI | PR | 95% CI | PR | 95% CI | PR | 95% CI |
| High Adult Child Educational Attainment,  Daughters | 0.92*** | 0.88 - 0.96 | 0.95** | 0.91 - 0.99 | 0.95** | 0.91 - 0.99 | 0.98 | 0.94 - 1.02 |
| High Adult Child Educational Attainment,  Sons | 0.98 | 0.94 - 1.02 | 0.98 | 0.94 - 1.03 | 0.95** | 0.92 - 1.00 | 0.96* | 0.93 - 1.00 |
| Source: Pooled observations of respondents in the SHARE Corona Survey, wave 1 and wave 2, of the Survey on Health, Aging and Retirement in Europe (SHARE) (N=30,471 for Daughters and N=30,992 for Sons). Notes: Exposure is a binary indicator of mean levels of ISCED-1997 classification for adult children, comparing those at or above the mean level to those below the mean level for all respondents in the same country. Controls include age, sex, educational attainment, nativity, country, marital status, parental education (mother and father), age of current spouse (if married/partnered) and educational attainment of current or former spouse, number of children and percentage of female children. *** *p* < 0.001, ** *p* < 0.01, * *p* < 0.05 | | | | | | | | |
|  |  |  |  |  |  |  |  |  |

| **eTable 6. Adult Child Educational Attainment and Older Parents' Psychosocial Outcomes During the COVID-19 Pandemic, by Parents' Sex** | | | | | | | | |
| --- | --- | --- | --- | --- | --- | --- | --- | --- |
|  |  |  |  |  |  |  |  |  |
|  | Feeling Nervous | | Feeling Depressed | | Sleep Problems | | Feeling Lonely | |
|  | PR | 95% CI | PR | 95% CI | PR | 95% CI | PR | 95% CI |
| Mothers | 0.95** | 0.91 - 0.99 | 0.96** | 0.92 - 1.00 | 0.95** | 0.91 - 0.99 | 1.00 | 0.96 - 1.04 |
| Fathers | 0.91*** | 0.85 - 0.97 | 0.91** | 0.85 - 0.98 | 0.92** | 0.86 - 0.98 | 0.91*** | 0.85 - 0.97 |
| Source: Pooled observations of respondents in the SHARE Corona Survey, wave 1 and wave 2, of the Survey on Health, Aging and Retirement in Europe (SHARE) (N=23,226 for Mothers and N=17,166 for Fathers). Notes: Exposure is a binary indicator of mean levels of ISCED-1997 classification for adult children, comparing those at or above the mean level to those below the mean level for all respondents in the same country. Controls include age, sex, educational attainment, nativity, country, marital status, parental education (mother and father), age of current spouse (if married/partnered) and educational attainment of current or former spouse, number of children and percentage of female children. *** *p* < 0.001, ** *p* < 0.01, * *p* < 0.05 | | | | | | | | |
|  |  |  |  |  |  |  |  |  |

| **eTable 7. Adult Child Educational Attainment and Older Parents' Worsened Psychosocial Outcomes Since the Pre-Pandemic Period** | | | | | | | | |
| --- | --- | --- | --- | --- | --- | --- | --- | --- |
|  |  |  |  |  |  |  |  |  |
|  | Perception of Worsened Feelings of  Nervousness | | Perception of Worsened Feelings of  Depression | | Perception of Worsened  Sleep Problems | | Perception of Worsened Feelings of  Loneliness | |
|  | PR | 95% CI | PR | 95% CI | PR | 95% CI | PR | 95% CI |
| High Adult Child Educational Attainment | 0.95* | 0.90 - 1.01 | 1.01 | 0.95 - 1.08 | 0.91* | 0.82 - 1.01 | 1.05 | 0.97 - 1.13 |
| Source: Respondents in the SHARE Corona Survey wave 1 of the Survey on Health, Aging and Retirement in Europe (SHARE) (N=20,916). Notes: Exposure is a binary indicator of mean levels of ISCED-1997 classification for adult children, comparing those at or above the mean level to those below the mean level for all respondents in the same country. Controls include age, sex, educational attainment, nativity, country, marital status, parental education (mother and father), age of current spouse (if married/partnered) and educational attainment of current or former spouse, number of children and percentage of female children. *** *p* < 0.001, ** *p* < 0.01, * *p* < 0.05 | | | | | | | | |
|  |  |  |  |  |  |  |  |  |

| **eTable 8. Adult Child Educational Attainment and Older Parents' Psychosocial Outcomes During the COVID-19 Pandemic, by Country-Level COVID-19 Intensity (Cases)** | | | | | | | | |
| --- | --- | --- | --- | --- | --- | --- | --- | --- |
|  |  |  |  |  |  |  |  |  |
|  | Perception of Worsened Feelings of  Nervousness | | Perception of Worsened Feelings of  Depression | | Perception of Worsened  Sleep Problems | | Perception of Worsened Feelings of  Loneliness | |
|  | PR | 95% CI | PR | 95% CI | PR | 95% CI | PR | 95% CI |
| High Adult Child Educational Attainment | 1.00 | 0.92 - 1.09 | 1.06 | 0.96 - 1.17 | 1.00 | 0.87 - 1.16 | 1.10* | 0.99 - 1.23 |
| High Adult Child Educational Attainment*  High Covid-19 Intensity | 0.90 ^a^ | 0.81 - 1.00 | 0.92 ^b^ | 0.81 - 1.04 | 0.82 ^c^ | 0.68 - 0.99 | 0.91 ^d^ | 0.79 - 1.05 |
| Source: Respondents in the SHARE Corona Survey wave 1 of the Survey on Health, Aging and Retirement in Europe (SHARE) (N=20,916). Notes: Exposure is the multiplicative interaction between a binary indicator of mean levels of ISCED-1997 classification for adult children, comparing those at or above the mean level to those below the mean level for all respondents in the same country, and a country-level COVID-19 intensity binary indicator of cases/1000 population contrasting countries at or above the median to those below the median of all included countries in the SCS. Data for the country-level COVID-19 intensity indicator comes from Our World in Data, sourced from Johns Hopkins University. Controls include age, sex, educational attainment, nativity, country, marital status, parental education (mother and father), age of current spouse (if married/partnered) and educational attainment of current or former spouse, number of children and percentage of female children. ^a.^p-value = 0.059, ^b.^p-value = 0.186, ^c.^p-value = 0.044, ^d.^p-value = 0.188. *** *p* < 0.001, ** *p* < 0.01, * *p* < 0.05 | | | | | | | | |
|  |  |  |  |  |  |  |  |  |

| **eTable 9. Adult Child Educational Attainment and Older Parents' Psychosocial Outcomes During the COVID-19 Pandemic, by Country-Level COVID-19 Intensity (Deaths)** | | | | | | | | |
| --- | --- | --- | --- | --- | --- | --- | --- | --- |
|  |  |  |  |  |  |  |  |  |
|  | Perception of  Worsened Feelings of  Nervousness | | Perception of Worsened Feelings of  Depression | | Perception of Worsened Sleep  Problems | | Perception  of Worsened Feelings of Loneliness | |
|  | PR | 95% CI | PR | 95% CI | PR | 95% CI | PR | 95% CI |
| High Adult Child Educational Attainment | 0.99 | 0.91 - 1.07 | 1.06 | 0.96 - 1.16 | 0.98 | 0.85 - 1.13 | 1.09 | 0.98 - 1.21 |
| High Adult Child Educational Attainment*  High Covid-19 Intensity | 0.92^a^ | 0.83 - 1.03 | 0.93^b^ | 0.82 - 1.05 | 0.85^c^ | 0.70 - 1.03 | 0.93^d^ | 0.80 - 1.07 |
| High Country-Level Covid-19 Intensity | | | | | | | | |
| High Adult Child Educational Attainment | 0.93* | 0.86 - 1.01 | 0.97 | 0.89 - 1.06 | 0.82*** | 0.71 - 0.95 | 1.01 | 0.91 - 1.12 |
| Low Country-Level Covid-19 Intensity | | | | | | | | |
| High Adult Child Educational Attainment | 0.96 | 0.90 - 1.02 | 1.02 | 0.95 - 1.10 | 0.97 | 0.92 - 1.04 | 1.02 | 0.96 - 1.07 |
| Source: Respondents in the SHARE Corona Survey wave 1 of the Survey on Health, Aging and Retirement in Europe (SHARE) (N=20,916). Notes: Exposure is the multiplicative interaction between a binary indicator of mean levels of ISCED-1997 classification for adult children, comparing those at or above the mean level to those below the mean level for all respondents in the same country, and a country-level COVID-19 intensity binary indicator of deaths/1000 population contrasting countries at or above the median to those below the median of all included countries in the SCS. Data for the country-level COVID-19 intensity indicator comes from Our World in Data, sourced from Johns Hopkins University. Controls include age, sex, educational attainment, nativity, country, marital status, parental education (mother and father), age of current spouse (if married/partnered) and educational attainment of current or former spouse, number of children and percentage of female children. ^a.^p-value = 0.147, ^b.^p-value = 0.238, ^c.^p-value = 0.099, ^d.^p-value = 0.294. *** *p* < 0.001, ** *p* < 0.01, * *p* < 0.05 | | | | | | | | |
|  |  |  |  |  |  |  |  |  |

| **eTable 10. Adult Child Educational Attainment and Older Parents' Worsened Psychosocial Outcomes Compared to the Pre-Pandemic Period, by Adult Child Sex** | | | | | | | | |
| --- | --- | --- | --- | --- | --- | --- | --- | --- |
|  |  |  |  |  |  |  |  |  |
|  | Perception of  Worsened Feelings of Nervousness | | Perception of  Worsened Feelings of Depression | | Perception of Worsened Sleep Problems | | Perception of Worsened Feelings of  Loneliness | |
|  | PR | 95% CI | PR | 95% CI | PR | 95% CI | PR | 95% CI |
| High Adult Child Educational Attainment, Daughters | 0.93** | 0.87 - 0.99 | 0.97 | 0.90 - 1.04 | 0.91 | 0.81 - 1.02 | 1.03 | 0.95 - 1.13 |
| High Adult Child Educational Attainment, Sons | 0.99 | 0.92 - 1.05 | 1.07* | 1.00 - 1.16 | 0.99 | 0.88 - 1.11 | 1.03 | 0.94 - 1.12 |
| Source: Respondents in the SHARE Corona Survey wave 1 of the Survey on Health, Aging and Retirement in Europe (SHARE) (N=15,777 for Daughters and N=16,046 for Sons). Notes: Exposure is a binary indicator of mean levels of ISCED-1997 classification for adult children, comparing those at or above the mean level to those below the mean level for all respondents in the same country. Controls include age, sex, educational attainment, nativity, country, marital status, parental education (mother and father), age of current spouse (if married/partnered) and educational attainment of current or former spouse, number of children and percentage of female children. *** *p* < 0.001, ** *p* < 0.01, * *p* < 0.05 | | | | | | | | |
|  |  |  |  |  |  |  |  |  |

| **eTable 11. Adult Child Educational Attainment and Older Parents' Worsened Psychosocial Outcomes Compared to the Pre-Pandemic Period, by Parents' Sex** | | | | | | | | |
| --- | --- | --- | --- | --- | --- | --- | --- | --- |
|  |  |  |  |  |  |  |  |  |
|  | Perception of  Worsened  Feelings of Nervousness | | Perception of  Worsened  Feelings of Depression | | Perception of  Worsened  Sleep Problems | | Perception of  Worsened  Feelings of Loneliness | |
|  | PR | 95% CI | PR | 95% CI | PR | 95% CI | PR | 95% CI |
| Mothers | 0.97 | 0.91 - 1.04 | 1.06 | 0.98 - 1.15 | 0.93 | 0.82 - 1.06 | 1.12** | 1.02 - 1.22 |
| Fathers | 0.90* | 0.81 - 1.01 | 0.91 | 0.80 - 1.04 | 0.85* | 0.71 - 1.02 | 0.89 | 0.77 - 1.04 |
| Source: Respondents in the SHARE Corona Survey wave 1 of the Survey on Health, Aging and Retirement in Europe (SHARE) (N=11,959 for Mothers and N=8,957 for Fathers). Notes: Exposure is a binary indicator of mean levels of ISCED-1997 classification for adult children, comparing those at or above the mean level to those below the mean level for all respondents in the same country. Controls include age, sex, educational attainment, nativity, country, marital status, parental education (mother and father), age of current spouse (if married/partnered) and educational attainment of current or former spouse, number of children and percentage of female children. *** *p* < 0.001, ** *p* < 0.01, * *p* < 0.05 | | | | | | | | |
|  |  |  |  |  |  |  |  |  |

| **eTable 12. Associations Between Adult Child Educational Attainment and Family Contact and Support During the COVID-19 Pandemic** | | | | | | |
| --- | --- | --- | --- | --- | --- | --- |
|  | Little/no contact  with children | | Received help from child(ren)  with basic necessities | | Helped child(ren)  with basic necessities | |
|  | PR | 95% CI | PR | 95% CI | PR | 95% CI |
| High Adult Child Educational Attainment | 1.07*** | 1.04 - 1.10 | 1.01 | 0.98 - 1.04 | 1.00 | 0.92 - 1.09 |
| Source: Pooled observations of respondents in the SHARE Corona Survey, wave 1 and wave 2, of the Survey on Health, Aging and Retirement in Europe (SHARE) (N=40,392 for Little/no contact with children; N=26,713 for Received help from child(ren) with basic necessities; N=20,638 for Helped child(ren) with basic necessities). Notes: Exposure is a binary indicator of mean levels of ISCED-1997 classification for adult children, comparing those at or above the mean level to those below the mean level for all respondents in the same country. Controls include age, sex, educational attainment, nativity, country, marital status, parental education (mother and father), age of current spouse (if married/partnered) and educational attainment of current or former spouse, number of children and percentage of female children. *** *p* < 0.001, ** *p* < 0.01, * *p* < 0.05 | | | | | | |

| **eTable 13. Associations Between Adult Child Educational Attainment and COVID-19 Related Outcomes During the COVID-19 Pandemic** | | | | | | |
| --- | --- | --- | --- | --- | --- | --- |
|  | Self or someone close  tested positive | | Self or someone close  hospitalized | | Someone close  Died | |
|  | PR | 95% CI | PR | 95% CI | PR | 95% CI |
| High Adult Child Educational Attainment | 1.10*** | 1.06 - 1.15 | 1.05 | 0.97 - 1.13 | 0.99 | 0.90 - 1.09 |
| Source: Pooled observations of respondents in the SHARE Corona Survey, wave 1 and wave 2, of the Survey on Health, Aging and Retirement in Europe (SHARE) (N=40,392). Notes: Exposure is a binary indicator of mean levels of ISCED-1997 classification for adult children, comparing those at or above the mean level to those below the mean level for all respondents in the same country. Controls include age, sex, educational attainment, nativity, country, marital status, parental education (mother and father), age of current spouse (if married/partnered) and educational attainment of current or former spouse, number of children and percentage of female children. *** *p* < 0.001, ** *p* < 0.01, * *p* < 0.05 | | | | | | |

| **eTable 14. Associations Between Adult Child Educational Attainment and COVID-19 Related Outcomes During the COVID-19 Pandemic** | | | | | | |
| --- | --- | --- | --- | --- | --- | --- |
|  | Little/no contact with children | | Received help from child(ren)  with basic necessities | | Helped child(ren)  with basic necessities | |
|  | PR | 95% CI | PR | 95% CI | PR | 95% CI |
| Continuous | 1.05*** | 1.03 - 1.06 | 1.01 | 0.99 - 1.02 | 0.99 | 0.95 - 1.04 |
| Quartiles |  |  |  |  |  |  |
| 1 (Reference category) |  |  |  |  |  |  |
| 2 | 1.01 | 0.97 - 1.05 | 1.04** | 1.00 - 1.08 | 1.11* | 0.99 - 1.26 |
| 3 | 1.06*** | 1.02 - 1.10 | 1.01 | 0.97 - 1.04 | 1.02 | 0.92 - 1.13 |
| 4 | 1.14*** | 1.09 - 1.20 | 1.04* | 1.00 - 1.10 | 1.06 | 0.92 - 1.22 |
| Source: Pooled observations of respondents in the SHARE Corona Survey, wave 1 and wave 2, of the Survey on Health, Aging and Retirement in Europe (SHARE) (N=40,392 for Little/no contact with children; N=26,713 for Received help from child(ren) with basic necessities; N=20,638 for Helped child(ren) with basic necessities). Notes: Exposure is a continuous measure of the average ISCED-1997 score (range: 0-6) that was not standardized to country-specific averages as well as a categorical variable based on country-specific quartiles of average adult child educational attainment. Controls include age, sex, educational attainment, nativity, country, marital status, parental education (mother and father), age of current spouse (if married/partnered) and educational attainment of current or former spouse, number of children and percentage of female children. *** *p* < 0.001, ** *p* < 0.01, * *p* < 0.05 | | | | | | |

| **eTable 15. Associations Between Adult Child Educational Attainment and COVID-19 Related Outcomes During the COVID-19 Pandemic** | | | | | | |
| --- | --- | --- | --- | --- | --- | --- |
|  | Self or someone close  tested positive | | Self or someone close  hospitalized | | Someone close  died | |
|  | PR | 95% CI | PR | 95% CI | PR | 95% CI |
| Continuous | 1.05*** | 1.03 - 1.07 | 1.03 | 0.99 - 1.06 | 1.01 | 0.97 - 1.06 |
| Quartiles |  |  |  |  |  |  |
| 1 (Reference category) |  |  |  |  |  |  |
| 2 | 1.11*** | 1.05 - 1.17 | 1.10* | 0.99 - 1.23 | 1.13* | 0.99 - 1.28 |
| 3 | 1.12*** | 1.07 - 1.17 | 1.09* | 0.99 - 1.19 | 1.04 | 0.93 - 1.17 |
| 4 | 1.18*** | 1.11 - 1.26 | 1.04 | 0.91 - 1.19 | 1.16* | 1.00 - 1.35 |
| Source: Pooled observations of respondents in the SHARE Corona Survey, wave 1 and wave 2, of the Survey on Health, Aging and Retirement in Europe (SHARE) (N=40,392). Notes: Notes: Exposure is a continuous measure of the average ISCED-1997 score (range: 0-6) that was not standardized to country-specific averages as well as a categorical variable based on country-specific quartiles of average adult child educational attainment. Controls include age, sex, educational attainment, nativity, country, marital status, parental education (mother and father), age of current spouse (if married/partnered) and educational attainment of current or former spouse, number of children and percentage of female children. *** *p* < 0.001, ** *p* < 0.01, * *p* < 0.05 | | | | | | |
