# Supplementary material for: Adult child educational attainment and older parents’ psychosocial outcomes during the COVID-19 pandemic
Source: BMC Public Health. 2024 Jul 31;24:2056. doi: 10.1186/s12889-024-19425-6 (PMC11289967; doi:10.1186/s12889-024-19425-6)
Supplement: Supplementary file 2 — Supplementary Material 2. [file 12889_2024_19425_MOESM2_ESM.docx]

**Adult child educational attainment and older parents’ psychosocial outcomes during the COVID-19 pandemic**

K. Renata Flores Romero,^a^ Yulin Yang,^a^ Sharon H. Green, ^b^ Sirena Gutierrez, ^a^ Erika Meza, ^a^ Jacqueline M. Torres ^a^

a. Department of Epidemiology & Biostatistics, UC San Francisco, San Francisco, CA

b. Department of Demography, UC Berkeley, Berkeley, CA

Corresponding Author:

Jacqueline M. Torres

550 16^th^ Street

San Francisco, CA 94143

Email: [Jacqueline.Torres@ucsf.edu](mailto:Jacqueline.Torres@ucsf.edu)

Phone: 415.317.3261

**Supplemental Appendix**

| **SHARE Corona Surveys (SCS) 2020** | **SHARE Corona Surveys (SCS) 2021** |
| --- | --- |
| *Support received from children* | |
| CAS021_  How often did the following people from outside your home help you to obtain necessities, compared to before the outbreak of Corona? Less often, about the same, or more often?  CAS021_1 Own children:  1. Less often  2. About the same  3. More often  99. Not applicable  -1. Don't know  -2. Refusal | CAS120_  Since the outbreak of corona, were you helped by the following people from outside your home to obtain necessities, e.g. food, medications, or emergency household repairs? Please answer yes or no to each category.  *IWER: Read out each relationship and check the appropriate answer.*  CAS120_1 Own children  1. Yes  5. No  -1. Don't know  -2. Refusal |
| *Support given to children* | |
| CAS011_  Compared to before the outbreak of Corona, how often did you help the following people from outside your home to obtain necessities: less often, about the same, or more often?  IWER: Read out each relationship and check the appropriate answer.  CAS011_1 Own children:  1. Less often  2. About the same  3. More often  99. Not applicable  -1. Don't know  -2. Refusal | CAS110_  Since the outbreak of corona, have you helped the following people outside your home to obtain necessities, e.g. food, medications, or emergency household repairs? Please answer yes or no to each category.  *IWER: Read out each relationship and check the appropriate answer.*  *If respondent does not have any living parents, children, grandchildren or relatives, code 'Not applicable'.*  CAS110_1 Own children:  1. Yes  5. No  99. Not applicable  -1. Don't know  -2. Refusal |
| Source: Survey on Health, Aging and Retirement in Europe (SHARE). COVID-19 Questionnaire for Telephone Interviews Wave 1 (Last update: June 2^nd^, 2020) and COVID-19 Questionnaire for Telephone Interviews Wave 2 (Last update: April 16, 2021). | |
